# Deep learning for regulatory genomics: a survey of models, challenges, and applications

**DOI:** 10.1093/bioadv/vbaf271

**Published:** 2025-10-31

**Authors:** Fathima Nuzla Ismail, Abira Sengupta, Shanika Amarasoma

**Affiliations:** Department of Mathematics, State University of New York at Buffalo, Buffalo, NY 14260, United States; School of Computing, University of Otago, Dunedin 9016, New Zealand; Independent Researcher, AI & Advanced Analytics, Colombo 00900, Sri Lanka

## Abstract

This research reviews recent advances in deep learning approaches tailored for regulatory genomics. It highlights how computational methods help decipher complex regulatory mechanisms within non-coding genomic regions across various tissues, emphasizing predictive applications such as transcription factor binding, chromatin accessibility, RNA processes, and RNA-protein interactions. The paper also discusses the evolution from traditional neural networks to advanced models like transformers and graph neural networks, considering three-dimensional genomic structures. Despite the promising performance, it acknowledges ongoing challenges like overfitting, biological variability, and limited dataset diversity. It emphasizes the urgent need for continued development of interpretable deep learning models to improve functional genomic annotation, underlining this task’s significance in the genomics field.

## 1 Introduction

**Figure 1.**
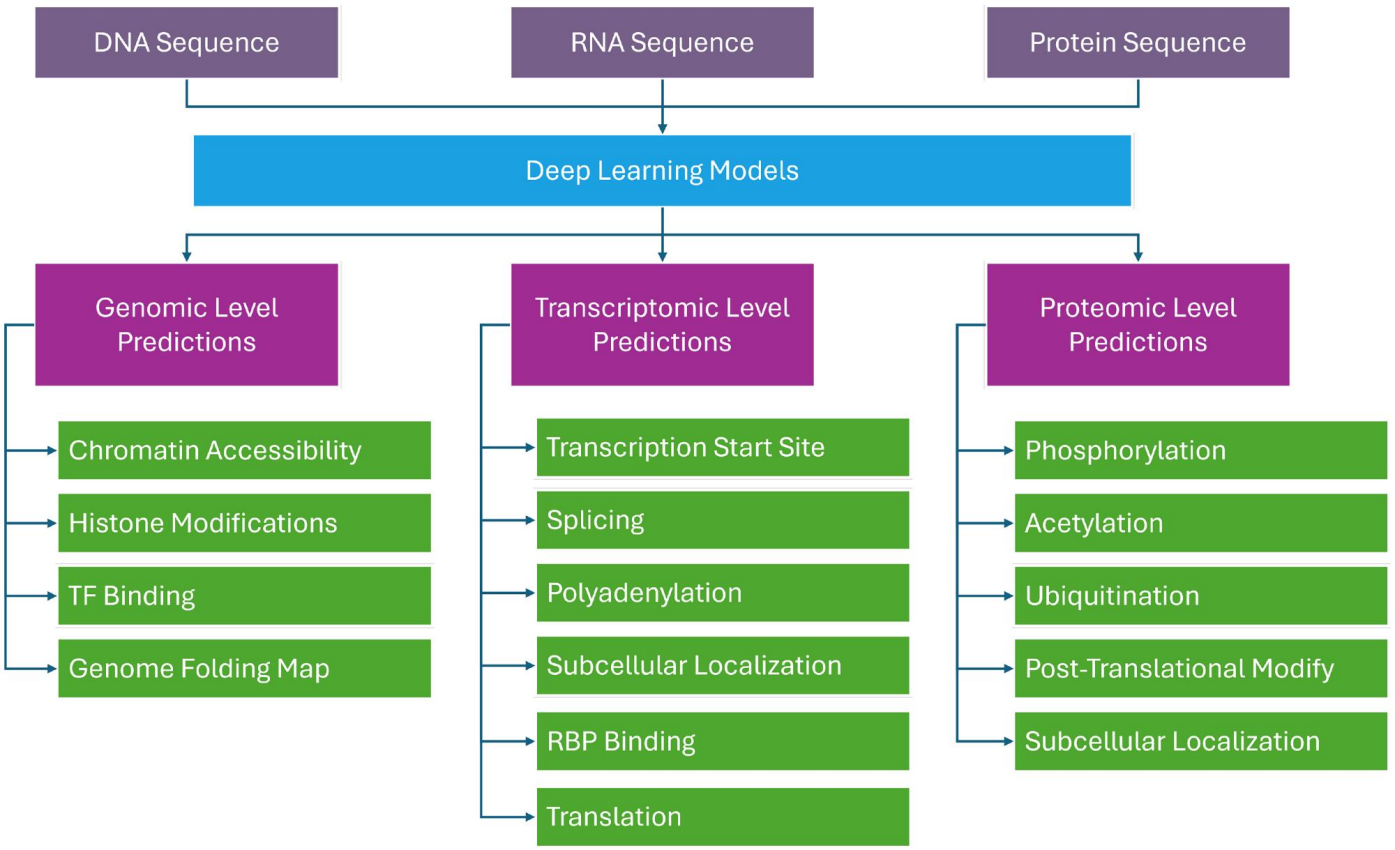
The figure illustrates an overview of using deep learning models to derive predictions at genomic, transcriptomic, and proteomic levels based on DNA, RNA, and protein sequences, respectively.

A key topic in cell biology is comprehending gene regulation, which takes place at the transcriptomic, proteomic, and genomic levels. Over the past two decades, breakthroughs in omics technologies—such as proteomics, transcriptomics, and genomics (Fig. 1)—have considerably improved our ability to examine gene control. Single-cell approaches, mass spectrometry, high-throughput sequencing, and microarrays have all made it possible to investigate regulatory systems at the fine scale. ChIP-seq (protein-DNA binding), CLIP-seq (Ule et al. 2005) (protein-RNA binding), DNase-seq and ATAC-seq (Buenrostro et al. 2013) (chromatin accessibility), RNA-seq (gene expression), and 30-READS (Hoque et al. 2013) (polyadenylation analysis) are now widely used methods.

These tools underpin large-scale projects like the 1000 Genomes Project, ENCODE, Roadmap Epigenomics, and GTEx, which aim to elucidate the biological pathways linking genotype to phenotype across individuals, species, and tissues. As omics datasets grow, so does the demand for analytical algorithms that can extract meaningful patterns. Traditionally, this has involved “shallow learning” methods—such as logistic regression (LR), support vector machines (SVM), and hidden Markov models (HMMs)—which depend on manually engineered features and predefined statistical models (Zeng and Lumley 2018). While effective, these models often fall short when prior knowledge is limited, as is frequently the case in omics. Their reliance on engineered features can reduce performance, limit discovery, and hinder model generalisability.

In contrast, learning algorithms that automatically extract discriminative features from data offer a compelling alternative. Deep learning models, in particular, can learn such features through training, typically via gradient descent optimization of a loss function (LeCun et al. 2015). These models use deep, hierarchical architectures—Deep Neural Networks (DNNs)—in which lower layers learn data-driven representations, while upper layers summarise them to perform inference. This makes deep learning especially promising for omics, where unknown patterns in biological sequences or measurements can be uncovered without relying on pre-engineered features.

The success of deep learning in omics is also due to the compatibility of biological data formats—such as DNA and RNA sequences—with models originally designed for natural language processing. For instance, regulatory grammar (i.e. interactions between sequence motifs) parallels linguistic structure, enabling deep learning to model complex regulatory codes (Alipanahi et al. 2015). This has led to practical applications and continues to inspire further exploration of deep learning’s role in decoding gene regulation.

In this survey, we provide a comprehensive overview of deep learning applications in gene regulation research using diverse omics data. We highlight applications across transcriptomic and genomic levels, explore various prediction tasks addressing biological questions, and examine model architectures and design principles. We also discuss emerging trends in integrating structural data, multi-omics profiles, and single-cell information. Our aim is to offer an up-to-date resource for researchers entering this exciting and rapidly evolving field.

As of 2025, the field has shifted toward foundation-scale, multimodal architectures such as AlphaGenome (Avsec et al. 2025), which unify genomic, epigenomic, and transcriptomic data within a single deep learning framework, reflecting the growing trend toward holistic modelling of gene regulation.

## 2 Neural networks used in gene regulation case studies

Over the past decade, neural network models in gene regulation studies have largely drawn inspiration from methods developed in natural language processing (NLP) and computer vision, where deep learning initially emerged. Early applications using tabular omics data frequently employed multi-layer perceptrons (MLPs), which consist of input layers that directly receive features from the dataset, one or more hidden layers that process this information, and an output layer that generates predictions.

Convolutional neural networks (CNNs), originally successful in image recognition and text classification (LeCun et al. 1998), have since proven valuable for analyzing raw biological sequences such as DNA, RNA, and proteins. CNNs use convolutional filters that preserve the spatial structure of sequential data, allowing efficient pattern recognition. More recently, transformers have become dominant in biological sequence analysis due to their success in NLP. Transformers effectively model pairwise relationships inside sequences without the inductive biases of CNNs or RNNs by using self-attention techniques. This has been quite successful in capturing the long-range dependencies that are essential for the regulation of genes. For example, the transformer-based model Enformer significantly outperforms CNN-based predecessors in predicting gene expression by attending to distal regulatory elements up to 100 kb away. This success has also paved the way for large-scale, self-supervised pretraining with models like DNABERT and its successors, which learn fundamental genomic grammar from unlabeled sequences before being fine-tuned for specific downstream tasks. These models mainly treat the genome as a linear sequence, but some have turned to 3D genomic structures, which are crucial for understanding gene regulation. For instance, GraphReg integrates 3D genomic data to enhance gene expression prediction. It utilises graph attention networks to model promoter-enhancer interactions, as defined by Hi-C data, explicitly. Because the graph structure explicitly stores long-range dependencies that are computationally difficult for traditional convolutional architectures to capture, this method enables GraphReg to perform better than sequence-only CNN models like Basenji on tasks requiring 3D context. Because the graph structure explicitly stores long-range dependencies that are computationally difficult for traditional convolutional architectures to capture, this method enables GraphReg to perform better than sequence-only CNN models like Basenji on tasks requiring 3D context.

**Figure 2.**
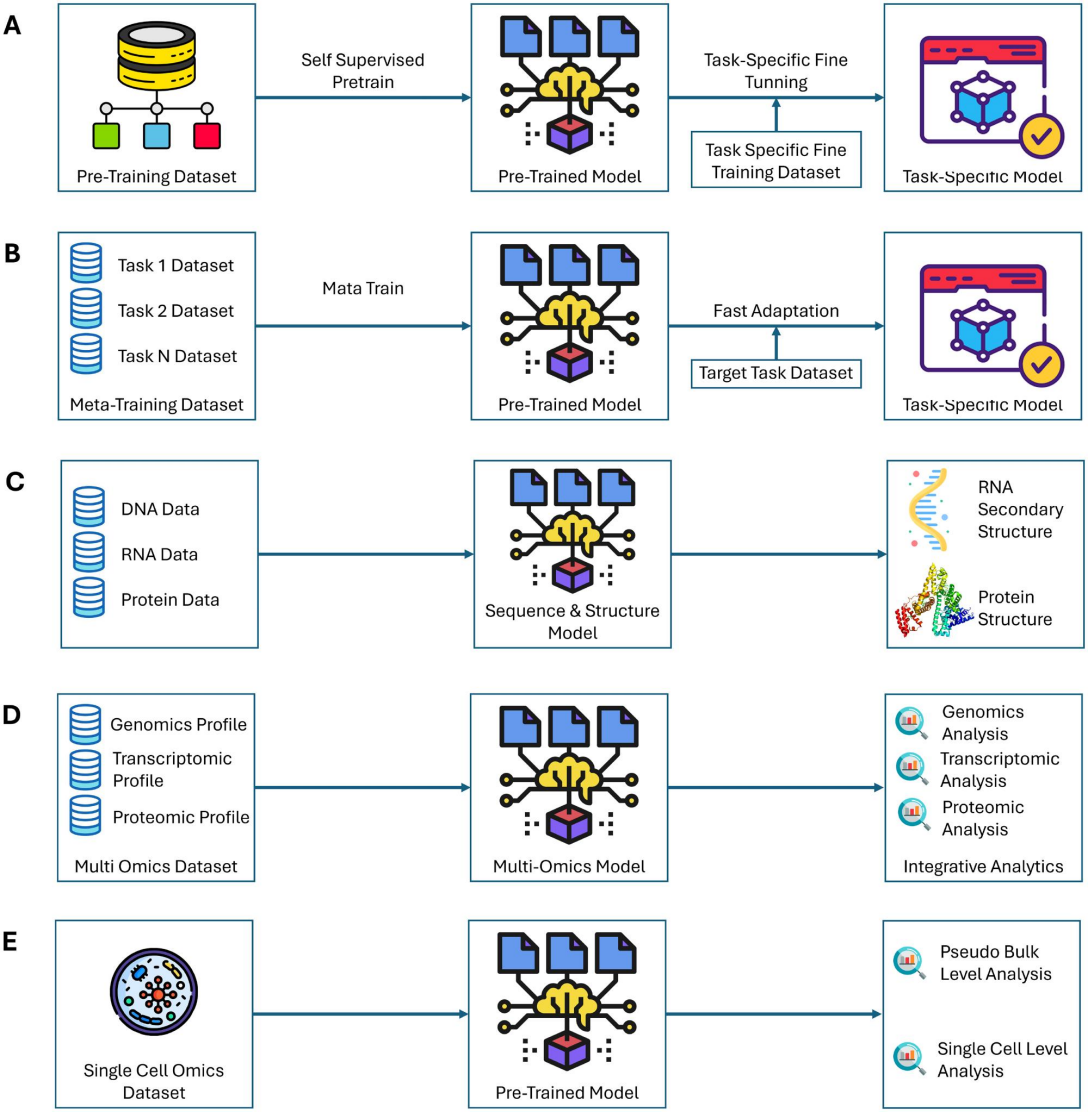
Next-generation deep learning approaches for gene regulation analysis.

Recurrent neural networks (RNNs), including long short-term memory (LSTM) units and gated recurrent units (GRUs), are particularly suited for biological sequence analysis due to their ability to model long-range dependencies through internal hidden states. Graph neural networks (GNNs) have also gained attention for processing structured biological data represented as graphs. Unlike MLPs that treat each data point independently, GNNs incorporate the dataset’s topology into the computation, allowing information propagation through graph-based hidden and output layers. More recently, transformers have become prominent in biological sequence analysis due to their success in NLP (Ji et al. 2021). Using self-attention mechanisms, transformers efficiently model pairwise relationships within sequences. They have also enabled self-supervised learning approaches in biology (Huang et al. 2010), a topic we discuss later in the paper (Fig. 2). To achieve optimal performance, efficiency, and biological interpretability, researchers often design deep learning architectures by combining or customizing one or more of these network types based on specific prediction tasks. While this article does not serve as an in-depth guide to neural network models, readers can refer to practical tutorials (Zou et al. 2019) and foundational deep learning textbooks (Fu et al. 2019) for further study.

## 3 Genomics used in gene regulation case studies

This section reviews key studies applying deep learning to genomic-level gene regulation. These approaches predict functional genomic characteristics from sequence data, framing the problem as supervised learning to understand how genetic variations affect regulatory mechanisms and to decode the genomic regulatory language. Tables 1 and 2 summarize these methodologies, focusing on their features, datasets, and underlying deep learning architectures. Many models are designed to predict species- and tissue/cell-type-specific genomic regulatory codes, as these vary across species and cell types. DeepBind (Alipanahi et al. 2015) was one of the first to predict nucleic acid-protein binding using CNNs trained on PBM, ChIP-seq, and HT-SELEX data. DeepSEA (Zhou and Troyanskaya 2015), another pioneer, uses CNNs to predict Deepsea predicts DNase I hypersensitivity (DHS), transcription factor TF binding, and histone modifications across multiple cell lines, trained on 125 DHS and 690 TF binding profiles from The ENCODE Project Consortium et al. (2012) and Roadmap Epigenomics (Bernstein et al. 2010). Its multi-task model can share learned genomic grammar to predict various signals, including expression quantitative trait loci (eQTLs) and functional non-coding mutations.

**Table 1.** Genomics-level deep-learning applications for expression modeling (2015–2025).

Method	Year	Main purpose	Key datasets	Model Arch.	Species	Ref
DeepBind	2015	TF/RBP binding prediction	PBMs, SELEX	CNN	Human	Alipanahi et al. (2015)
DeepSEA	2015	Chromatin feature + variant effect	ENCODE, Roadmap	CNN	Human	Zhou and Troyanskaya (2015)
Basset	2016	Chromatin accessibility	DNase-seq	CNN	Human	Kelley et al. (2016)
DanQ	2016	DNA function prediction	ENCODE	CNN + BiLSTM	Human	Quang and Xie (2016)
CpGenie	2017	DNA methylation prediction	RRBS, WGBS	CNN	Human	Zeng and Gifford (2017)
De-Fine	2018	TF binding site prediction	ENCODE ChIP-seq	CNN	Human	Wang et al. (2018)
Basenji	2018	Long-range regulatory activity	ENCODE	CNN w/dilation	Human	Kelley et al. (2018)
Expecto	2018	Gene expression prediction	GTEx, ENCODE	CNN + regression	Human	Zhou et al. (2018)
HiCPlus	2018	Hi-C resolution enhancement	Hi-C	CNN	Human	Zhang et al. (2018)
MMSplice	2018	Predict alternative splicing	GTEx	Modular NN	Human	Cheng et al. (2019)
AttentiveChrome	2018	Expression from histone marks	Epigenome datasets	RNN + Attention	Human	Singh et al. (2017)
DeepCAPE	2019	Enhancer prediction	Enhancer, ATAC-seq	Multi-modal CNN	Human	Chen et al. (2021)
DeepHistone	2019	Histone pattern prediction	ChIP-seq	CNN + RNN	Human	Yin et al. (2019)
DeepHiC	2019	Hi-C super-resolution	Hi-C	GAN	Human	Hong et al. (2020)
DeepATAC	2019	Regulatory activity prediction	ATAC-seq, ChIP-seq	CNN	Human	Hiranuma et al. (2017)
Selene	2019	DL framework for genomics	Customizable	CNN-based	Any	Chen et al. (2019)
Xpresso	2020	Expression from promoter DNA	Promoter seqs	CNN	Human	Agarwal and Shendure (2020)
DeepMEL	2020	Enhancer activity in melanoma	Melanoma ATAC/ChIP	CNN	Human	Minnoye et al. (2020)
DeepTACT	2020	Predict chromatin contacts	Hi-C, ATAC-seq	CNN + RNN	Human	Li et al. (2019)
Akita	2020	Predict 3D genome folding	Hi-C	CNN (2D dilation)	Human	Fudenberg et al. (2020)
DNABERT	2021	Pretrained transformer on DNA k-mers	Human genome	BERT	Human	Ji et al. (2021)
Enformer	2021	Long-range expression + epigenetics	Human, Mouse genomes	CNN + Transformer	Human, Mouse	Avsec et al. (2021a)
BPNet	2021	Base-resolution TF binding	ChIP-nexus	CNN w/dilation	Human	Avsec et al. (2021b)
Orca	2022	Multi-scale 3D genome prediction	Hi-C	Hierarchical CNN	Human	Zhou (2022)
GraphReg	2022	Expression from 3D interactions	Hi-C, RNA-seq	Graph NN	Human	Karbalayghareh et al. (2022)
EpiBERTope	2022	Epitope prediction	Epitope datasets	Transformer (BERT)	Human	Park et al. (2022)
Nucleotide Transformer	2023	Large-scale pre-trained transformer trained on diverse genomes for downstream genomic prediction tasks	Hi-C, ATAC-seq	Transformer (BERT)	Human	Dalla-Torre et al. (2025)
HyenaDNA	2023	Long-range genomic modeling using the Hyena operator for efficient scaling beyond transformers	CNN	Hi-C, ATAC-seq	Human	Nguyen et al. (2023)
EpiGePT	2023	Transformer-based multi-omics model integrating sequence, chromatin accessibility, and histone modifications for gene regulation prediction	Hi-C, ATAC-seq, histone modification ChIP-seq	Transformer (BERT)	Human	Gao et al. (2024)
AlphaGenome	2025	Foundation model integrating multi-omics and spatial genomics for regulatory element discovery	ENCODE, GTEx, single-cell ATAC-seq	Multimodal Transformer	Human	Avsec et al. (2025)
GeneFormer2	2025	Self-supervised transformer pre-trained on single-cell RNA and chromatin data	scRNA-seq, scATAC-seq	Transformer	Human, Mouse	Ito et al. (2025)

**Table 2.** Transcriptomic-level deep-learning applications for expression modeling (2015–2025).

Method	Year	Main Purpose	Key Datasets	Model Arch.	Species	Reference
MiRTDL	2015	microRNA target prediction	miRTarBase, TargetScan	Deep neural net	Human	Cheng et al. (2016)
CNNProm	2017	Promoter prediction from sequence	Promoter DBs	CNN	Human	Umarov and Solovyev (2017)
DeeReCT-PromID	2019	Promoter identification	FANTOM5, EPD	CNN + RNN	Human	Umarov et al. (2019)
DeeReCT-PolyA	2019	Predict poly(A) site usage	PolyA_DB, ENCODE	CNN + BiLSTM	Human	Xia et al. (2019)
DeeReCT-APA	2019	Alternative polyadenylation prediction	3′UTR sequences	CNN	Human	Li et al. (2022)
RNATracker	2019	Predict RNA subcellular localization	RNALocate, lncATLAS	BiLSTM + Attention	Human	Yan et al. (2019)
APARENT	2019	Polyadenylation site usage modeling	Synthetic	CNN	Human	Bogard et al. (2019)
NucleicNet	2019	RNA-binding protein interaction prediction	RBP datasets (e.g. CLIP-seq)	Residual CNN	Human	Lam et al. (2019)
DARTS	2019	Alternative splicing prediction	GTEx, ENCODE	BiLSTM + Attention	Human	Zhang et al. (2019)
SpliceAI	2019	Splice variant effect prediction	Genomic annotations	Deep ResNet	Human	Jaganathan et al. (2019)
DeeReCT-TSS	2021	TSS prediction	FANTOM5, RefSeq	CNN + BiLSTM	Human	Zhou et al. (2022)
Pangolin	2022	Predict tissue-specific splicing from variants	GTEx, 5’SS/3’SS features	CNN + Residual blocks	Human	Zeng and Li (2022)
scBERT	2023	Transformer-based model for single-cell transcriptomics, enabling cell type and splicing prediction across tissues	RNA-seq	Deep neural net	Human	Yang et al. (2022)
RNA-Mamba	2024	State-space model adapted for RNA splicing and secondary structure prediction at scale	RNA-seq	Deep neural net	Human	Fradkin et al. (2025)

Following DeepSEA’s success, other models, such as Basset (Kelley et al. 2016), built on CNNs for better DHS prediction, while DanQ (Quang and Xie 2016) combined CNNs with bidirectional LSTMs, outperforming DeepSEA on the same datasets. CpGenie (Zeng and Gifford 2017) predicts DNA methylation states, while De-Fine (Wang et al. 2018) focuses on TF binding prediction using cell-line-specific genomes, demonstrating the importance of genome diversity.

Basenji (Andlauer et al. 2016) extends CNNs to analyze longer genomic sequences by using dilated convolutions, enabling it to capture long-range dependencies. Expecto (Zhou et al. 2018) further builds on DeepSEA, expanding input size and incorporating regression for gene expression predictions using GTEx and Roadmap Epigenomics data.

DeepMEL (Agarwal and Shendure 2020) predicts chromatin accessibility in melanoma using TAC-seq data and identifies co-accessible regions, while BPNet (Avsec et al. 2021b) identifies cooperativity between TF motifs. These models mainly treat the genome as a linear sequence, but some have turned to 3D genomic structures, which are crucial for understanding gene regulation.

Akita Fudenberg et al. predicts genome folding, a key aspect of the genome’s 3D structure, using Hi-C and Micro-C data. Orca (Zhou 2022) builds on Akita, predicting genome folding at varying resolutions using 2D CNNs and multi-resolution sequence encoders, incorporating DHS and histone modification data for multi-functional predictions.

GraphReg (Karbalayghareh et al. 2022) integrates 3D genomic data to enhance gene expression prediction, using graph attention networks for Seq-GraphReg (based on genomic sequences and 3D data) and Epi-GraphReg (using epigenetic and 3D profiles for tissue-agnostic predictions).

**Recent Developments (2025)** In early 2025, several large-scale foundation models extended the scope of genomic deep learning. AlphaGenome (Avsec et al. 2025) introduced a multimodal transformer architecture that integrates sequence, chromatin accessibility, and 3D genomic data across diverse tissues, significantly improving cross-species generalisation in enhancer and promoter prediction. Similarly, GeneFormer2 (Ito et al. 2025) leveraged self-supervised pretraining on millions of single-cell expression and accessibility profiles, achieving state-of-the-art accuracy in predicting gene regulatory interactions. These models represent a new generation of scalable foundation frameworks in regulatory genomics, paralleling advances in protein and molecular modelling.

## 4 Transcriptomic in gene regulation case studies

RNA splicing is a key process contributing to the diversity of the eukaryotic transcriptome, allowing for the generation of mRNAs with different 3′ untranslated regions (30 UTRs) through the use of multiple polyadenylation sites (PASs). These 30 UTRs contain regulatory features, and the final location of the mRNA, determined by its subcellular RNA location, influences the spatial distribution of newly transcribed mRNAs. MicroRNAs (miRNAs) can target mRNAs to suppress specific gene production. The mRNA’s 5′ UTR, which affects its translational efficiency, significantly regulates protein synthesis rates. Table 3 outlines the challenges in applying deep learning to transcriptomic predictions. Although some methods discussed in the genomics section also include transcriptomic-level predictions, the power of deep learning in transcriptomics is increasingly evident. Early deep learning-based models for transcriptomic regulation focused on promoter recognition and splicing prediction.

**Table 3.** Key challenges in deep learning and corresponding solutions.

Method name	Year	Description	Challenges
**Enhancements in Stochastic Gradient Descent (SGD) Optimization:**			
Adagrad Duchi et al. (2011)	2011	Adapts learning rate per parameter automatically	Optimization difficulties in deep models
RMSProp Tieleman and Hinton (2012)	2013	Maintains a moving average of squared gradients	Optimization difficulties in deep models
Adam Kingma and Ba (2015)	2014/2015	Combines adaptive learning rates and momentum	Optimization difficulties in deep models
NAdam Dozat (2016)	2016	Enhances Adam with Nesterov momentum	Optimization difficulties in deep models
AdamW Loshchilov and Hutter (2019)	2017	Separates weight decay from gradient update	Optimization difficulties in deep models
RAdam Liu et al. (2020)	2019	Rectifies variance in adaptive learning	Optimization difficulties in deep models
AdaBelief Zhuang et al. (2020)	2020	Provides fast, stable convergence with belief tracking	Optimization difficulties in deep models
MadGrad Defazio and Jelassi (2021)	2021	Momentum-driven gradients with robust results	Optimization difficulties in deep models
**Hyperparameter Tuning Tools:**			
RayTune Liaw et al. (2018)	2018	Distributed tuning for PyTorch and TensorFlow	Hyperparameter tuning inefficiency
KerasTuner O’Malley et al. (2019)	2019	Keras-compatible tool for hyperparameter search	Hyperparameter tuning inefficiency
Optuna Akiba et al. (2019)	2019	Lightweight tuner with pruning and sampling strategies	Hyperparameter tuning inefficiency
Ax (Facebook) Bakshy et al. (2018)	2020	Bayesian optimization and adaptive experimentation	Hyperparameter tuning inefficiency
**Interpretability Techniques for Models:**			
Perturbation-Based Methods	2014	Observe output change when input is altered	Lack of interpretability
		Example: Occlusion tests Zeiler and Fergus (2014)	
Attribution-Based Methods	2013	Assign relevance scores to input features	Lack of interpretability
Integrated Gradients Sundararajan et al. (2017)	2017	Uses path-integral to estimate feature impact	Lack of interpretability methods
SHAP Lundberg and Lee (2017)	2017	Feature importance via Shapley value theory	Lack of interpretability methods
DeepLIFT Shrikumar et al. (2017)	2017/2018	Compares activations to a reference input	Lack of interpretability methods
Captum Kokhlikyan et al. (2020)	2020	PyTorch library for interpretability	Lack of interpretability methods
Grad-CAM Selvaraju et al. (2020)	2019	Highlights class-relevant regions in CNNs	Lack of interpretability
Model-Based Methods	2019	Build interpretable models using design constraints	Lack of interpretability
		Example: Bottleneck layers, attention mechanisms Jain and Wallace (2019)	

### 4.1 Promoter recognition

CNNProm (Umarov and Solovyev 2017), one of the first deep learning models for identifying promoter sequences, employed one to two CNN layers to classify sequences into promoter/non-promoter categories. It was effective across prokaryotes and eukaryotes (human, mouse, Arabidopsis). DeeReCT-PromID (Umarov et al. 2019), an extension of CNNProm, expanded input size to 600 bp for genome-wide scanning and introduced a novel approach to selecting complex negative examples to reduce false-positive rates, as most genomic areas are non-promoters. DeeReCT-TSS further improved this by integrating RNA-seq data to infer promoter usage in various cell lines, trained on 10 FANTOM5 cell lines with matched CAGE-seq data (Noguchi et al. 2017).

### 4.2 RNA splicing

RNA splicing, which generates diversity by combining different exon and intron pairings, plays a vital role in transcriptomic regulation. Early deep learning models (Fagnani et al. 2007) used a one-layer neural network to predict cassette exon usage across various mouse tissues, using a 1014-dimensional vector as input. Leung et al. (2014) expanded this model, increasing sequence features to 1393 dimensions and using 11019 mouse cassette exons to predict tissue-specific splicing patterns. This was a major breakthrough, which replicated for human cassette exons using similar methods.

### 4.3 Alternative splicing

DARTS (Zhang et al. 2019) combined low-coverage RNA-seq with deep learning and RNA-binding protein (RBP) expression data to predict differential cassette exon usage, enabling splicing analysis even with insufficient RNA-seq data. SpliceAI (Jaganathan et al. 2019) was the first deep learning-based model to predict splice sites for all splicing types directly from raw pre-mRNA sequences. It uses residual blocks and dilated convolutions to handle lengthy pre-mRNA sequences (up to 5000 nt) for splice site prediction. Pangolin (Zeng and Li 2022) expanded SpliceAI’s capabilities into a multi-task framework, detecting splice sites across four tissue types and four species.

### 4.4 Polyadenylation and PAS prediction

Polyadenylation, which adds a poly(A) tail to mRNAs and determines transcription termination, is also regulated by PASs. DeeReCT-PolyA (Xia et al. 2019) used a CNN to predict PASs in human and mouse datasets, outperforming non-deep-learning techniques. Leung et al. (2018) used a two-branch CNN for PAS quantification, comparing competing PASs of a gene. DeeReCT-APA (Li et al. 2022) combined CNN and Bilstm architectures to handle multiple PASs per gene, surpassing previous models in simulating the interactions between competing PASs. APARENT (Bogard et al. 2019) trained a two-layer CNN on 3 million synthetic MPRA sequences to predict PAS regulatory activity and optimized PAS sequences for desired regulatory outcomes.

### 4.5 mRNA localisation

mRNA subcellular localisation, both physical (in various subcellular structures) and quantitative (adjusting mRNA accessibility to ribosomes), plays a crucial role in regulating gene expression. RNATracker (Yan et al. 2019) is a deep learning model that classifies mRNA into its likely subcellular locations using sequence and secondary structure data. The model, trained on APEX-RIP and CeFra-Seq data, classifies mRNA into categories like cytosol, nucleus, or membrane.

### 4.6 MiRNA-MRNA interactions

MiRNAs regulate mRNA expression post-transcriptionally. MiRTDL (Cheng et al. 2016), a CNN-based model, predicts possible miRNA-mRNA interactions, an essential component of transcriptomic regulation. Regulatory Features in 5′ UTRs and Translational Efficiency: The 5′ UTR of mRNA significantly influences ribosomal translational efficiency. Cuperus et al. (2017) created a three-layer CNN to predict translational efficiency based on 5′ UTR sequences. The model was trained on 489 348 synthesized 5′ UTRs in yeast, helping predict the efficiency of protein synthesis.

### 4.7 RNA-protein interactions

DeepBind (Alipanahi et al. 2015) also predicts RNA-protein bindings, using sequence data to model interactions across 24 eukaryotes. NucleicNet (Lam et al. 2019) takes a different approach, predicting RNA-binding protein (RBP) interactions based on the structures of ribonucleoproteins from the PDB (Berman et al. 2000). For an in-depth review of deep learning applications in RNA-protein binding, readers can refer to Wei et al. (2022).

### 4.8 Limitations of existing deep-learning applications in gene regulation

Despite recent advances, the application of deep learning in gene regulation is constrained by both general machine learning challenges and domain-specific issues. One prominent issue is overfitting, where models exhibit strong performance on benchmark datasets but fail to generalise to unseen data. This problem is especially critical in gene regulation due to several complex and persistent limitations:

- **Limited data availability in biological research** can hinder the development of robust machine learning models. Only a small portion of the genome, transcriptome, or proteome is typically relevant for any given regulatory event. For example, promoter sequences that govern transcription start sites (TSSs) are confined to specific genomic regions at the start of each gene, which inherently limits the variety of training data.
- **Biological and technical variability** across experimental conditions may further constrain a model’s ability to generalise. For instance, in the Basenji study, biological replicates—even within the same consortium—had an average Pearson correlation of only 0.479, making it difficult to distinguish between true generalisation and overfitting to experimental noise.
- **Dependence on endogenous sequences** raises concerns about the model’s generalisability. Many deep-learning models such as DeepSEA (Zhou and Troyanskaya 2015), Basset (Kelley et al. 2016), and ExPecto (Zhou et al. 2018) are trained solely on endogenous sequences from the human reference genome (e.g. GRCh37). This limits their applicability to novel variants that may follow a different regulatory grammar.
- **Lack of exogenous sequence data** further restricts the training scope. Only a few models, such as Cuperus et al. (2017) for 5′ UTR translation efficiency and APARENT (Bogard et al. 2019) for polyadenylation, utilised measurements from exogenous sequences generated via MPRA data. This highlights the urgent need for more diverse training datasets in gene regulation modeling.
- **Model interpretability** is a crucial threshold in the application of deep learning to regulatory genomics. Interpretability allows models to uncover mechanisms and regulatory codes that are embedded in DNA and RNA sequences, in addition to their predictive performance. To find tissue-specific transcription factor motifs and evaluate their relative contributions to gene expression, attribution-based techniques like Integrated Gradients, SHAP, and DeepLIFT have been employed. Similarly, long-range dependencies that influence chromatin accessibility across species have been brought to light by attention processes in transformer models like DNABERT and Enformer. Perturbation-based techniques, including in silico mutagenesis, have further allowed researchers to test variant effects directly, generating experimentally verifiable hypotheses. Deep learning models are transformed from black-box predictors into tools for generating hypotheses that can shed light on the regulatory processes specific to various cell types and how they differ across tissues and species, thanks to these interpretability frameworks.

To complement the qualitative advances discussed above, Table 4 summarizes comparative performance metrics reported across representative deep learning models for genomic prediction tasks. These benchmarks highlight how architectural innovations—from early convolutional models like DeepSEA and Basset to transformer- and graph-based frameworks such as Enformer and GraphReg—have progressively improved predictive accuracy while enabling more interpretable and biologically grounded inferences.

**Table 4.** Comparative performance of key genomic models on selected tasks.

Model	Year	Prediction Task	Key Metric	Reported Score	Key Strength
DeepSEA	2015	TF Binding	AUPRC (avg)	0.91	Pioneering multi-task CNN for chromatin effects
Basset	2016	Chromatin Accessibility	AUPRC	0.69	Deeper CNN architecture for DHS prediction
DanQ	2016	DNA Function	AUPRC (avg)	0.93	Hybrid CNN-RNN captures motifs & syntax
Basenji	2018	Gene Expression	Pearson’s r	0.67	Dilated CNNs for capturing long-range effects
Enformer	2021	Gene Expression	Pearson’s r	0.85	Transformer architecture models very long-range interactions
GraphReg	2022	Gene Expression	Pearson’s r	0.82	Graph NN explicitly models 3D interactions

Overfitting is a common problem when models perform well on benchmark datasets but are unable to generalize to new data. This issue is critical when it comes to gene control. For example, a model trained exclusively on ENCODE data for a specific transcription factor might inadvertently learn technical artifacts or batch effects present in the training set. The model’s performance can drastically decline when used on data for the same factor produced by a different consortium using marginally different techniques, demonstrating a failure to identify the actual biological signal.

Addressing these limitations requires a multi-faceted approach:

- **Data Augmentation:** Techniques such as sequence shifting, reversal, and random nucleotide substitutions can be used to generate synthetic training instances in order to overcome a lack of training data, which forces the model to acquire more resilient characteristics.
- **Advanced Regularisation:** Beyond standard methods like dropout, strategies such as adversarial training can improve generalization. This involves training the model on minimally perturbed examples to maximize prediction error, thereby making the model more robust to novel genetic variants.
- **Cross-Consortium Validation:** Systematically training on data from one project (e.g. ENCODE) and testing on another (e.g. Roadmap Epigenomics) is crucial for assessing true cross-context generalizability and moving beyond leaderboard-driven performance metrics.

In summary, overfitting, data scarcity, biological variability, and limited sequence diversity collectively pose significant barriers to the successful deployment of deep learning models in gene regulation. Addressing these limitations will be essential for developing models with true biological interpretability and cross-context generalisability.

## 5 Discussion

The development and evaluation of deep learning models in regulatory genomics increasingly rely on reproducible and scalable computational environments. Workflow orchestration frameworks such as Nextflow (Di Tommaso et al. 2017) and Snakemake (Köster and Rahmann 2012), as well as containerized solutions such as Mars (Ismail and Amarasoma 2025), have emerged to streamline bioinformatics pipelines and provide a reliable, secure environment. These tools encapsulate dependencies and standardize execution across heterogeneous computing systems, ensuring that preprocessing, model training, and evaluation steps can be reproduced and shared across studies. By encapsulating dependencies and standardizing execution across heterogeneous computing systems, these tools have emerged to streamline bioinformatics pipelines, instilling confidence in the research process.

Researchers can learn more about the mechanisms underlying gene regulation by including interpretability techniques in model creation. For instance, BPNet (Avsec et al. 2021b) demonstrated cooperative binding syntax amongst factors in addition to predicting base-resolution transcription factor binding. Likewise, DeepMEL’s cross-species enhancer study revealed both divergent and conserved enhancer logic in many tissues. These examples underscore the potential of interpretable models to provide biologically meaningful discoveries, such as tissue- or cell-type-specific regulation, and to uncover how regulatory mechanisms vary across species.

## Supplementary Material

vbaf271_Supplementary_Data
